# The role of histological subtypes in the survival of patients diagnosed with cutaneous or mucosal melanoma in the United States of America

**DOI:** 10.1371/journal.pone.0286538

**Published:** 2023-06-05

**Authors:** Mohammad A. Tabatabai, Nader Bahri, Patricia Matthews-Juarez, Donald Alcendor, Robert Cooper, Paul Juarez, Aramandla Ramesh, Niki Tabatabai, Karan P. Singh, Derek Wilus

**Affiliations:** 1 Meharry Medical College, Nashville, TN, United States of America; 2 University of California Los Angeles, Los Angeles, CA, United States of America; 3 University of Texas Health Sciences Center at Tyler, Tyler, TX, United States of America; The University of Sydney, AUSTRALIA

## Abstract

**Background:**

Literature presents limited information on histological subtypes and their association with other factors influencing the survival of melanoma patients. To explore the risk of death due to melanoma associated with histological subtypes, this retrospective study used the Surveillance, Epidemiology, and End Results program (SEER) data from 1998 to 2019.

**Methods:**

A total of 27,532 patients consisting of 15,527 males and 12,005 females. The Hypertabastic Accelerated Failure Time model was used to analyze the impact of histology on the survival of patients with cutaneous or mucosal melanoma.

**Results:**

The median survival time (MST) for cutaneous patients was 149 months, whereas those diagnosed with mucosal melanoma was 34 months. Nodular melanoma had a hazard ratio of 3.40 [95% CI: (2.94, 3.94)] compared to lentigo maligna melanoma. Across all histological subtypes, females had a longer MST, when compared to males. The hazard ratio (HR) of distant to localized melanoma was 9.56 [95% CI: (7.58, 12.07)].

**Conclusions:**

Knowledge of patients’ histological subtypes and their hazard assessment would enable clinicians and healthcare providers to perform personalized treatment, resulting in a lower risk of complication and higher survivability of melanoma patients. Significant factors were stage of the disease, age, histology, sex, and income. Focus should be placed on high-risk populations with severe and aggressive histological subtypes. Programs that emphasize preventive measures such as awareness, education, and early screening could reduce risk.

## Introduction

Analysis of melanoma patients’ survival probability, especially the magnitude and the influence of prognostic factors, as well as the connections between risk factors, can provide invaluable insights into opportunities and priorities for the design of an optimal and effective prevention modality or treatment strategy to save lives. Our comparative risk assessment provides an analytical framework to perform a comprehensive assessment of survival of patients diagnosed with different histological subtypes of melanoma. According to the American Cancer Society, melanoma accounts for about one percent of all skin cancers, but is the cause of the majority of skin cancer deaths. The incidence rate of melanoma is significantly higher in non-Hispanic Whites, hereafter referred to as Whites, when compared with non-Whites. Risk factors for melanoma include but are not limited to excessive exposure to natural or non-natural infrared rays, age, sex, race/ethnicity, presence of unusual moles, immune suppression, and family history of melanoma [1].

According to the American Cancer Society, the predicted number of new cases of cutaneous melanoma for the year 2022 is 57,180 among males and 42,600 among females. The predicted number of deaths for the same period is 5,080 among males and 2,570 among females. Between 2014–2018, the melanoma death rates for males and females were 3.4 and 1.4 per 100,000 respectively [2].

A vast majority of melanoma patients are diagnosed with superficial spreading melanoma. Cutaneous melanoma is among the most aggressive and fatal forms of malignant skin cancer. Early diagnosis is essential for better survival probability [3]. Superficial spreading melanoma and nodular melanoma cover the majority of cutaneous melanomas [4–7]. Susok et al. compared prognostic factors associated with nodular melanoma to superficial spreading melanoma, highlighting the need to compare histological factors [8]. A number of previous studies have examined melanoma survival based on histological subtypes and location. Schaule et al. evaluated the survival of people diagnosed with melanoma and brain metastases who underwent stereotactic radiotherapy and concurrent targeted therapy or immunotherapy [9]. Feng et al. observed differences in lifetime risks of cutaneous melanoma, by five histological subtypes and race/ethnicity in the United States [10]. Uehara et al. (2009) applied a Cox proportional hazards model to evaluate prognostic factors in Japanese patients with localized and invasive melanoma [11]. Previous studies used Cox regression and found significant differences in the survival probabilities of patients with a limited number of histological subtypes [12,13].

Abdel-Rahman (2019) used the Kaplan–Meier method to assess the impact of socioeconomic status on overall survival and multivariable Cox regression model to investigate such an impact on melanoma-specific survival [14]. Moore et al. used multivariable Cox regression to obtain hazard ratios (HR) and 95% confidence intervals for the survival of patients with malignant melanoma [15]. Xue et al. developed a Cox model based prognostic gene model to predict the prognosis of uveal melanoma patients [16]. A patients’ histological subtype must be included as additional information to the healthcare provider in order for them to reach a more accurate prognosis [17]. Cherobin et al. analyzed the effect of histopathological and epidemiological factors on the advancement of metastases in melanoma patients [18]. Frinton et al. demonstrated the influence of prognostic factors for melanoma patients with brain metastases [19].

The aim of this retrospective study is to explore the influence histological subtypes have in the survival of melanoma patients and the variations in the risk of death with regard to age, race, sex, income, region, and stage. The primary outcome is time from diagnosis of melanoma until death.

## Methods

This study analyzed the survival of patients diagnosed with melanoma using data from the Surveillance, Epidemiology, and End Results program (SEER) [20] from 1998 to 2019. Internal Review Board committee at Meharry Medical College granted approval to this research with an exempt status. Data was collected from the SEER*STAT software version 8.4.0. Race was categorized into two groups: Whites and non-Whites. Non-Whites are composed of African American/Blacks, Asian/Pacific Islanders, and American Indian/Alaskan. The variable stage of the disease consists of localized, regional, and distant (metastatic). Income, initially grouped into 10 categories by SEER, was refined into the following four categories: individuals making less than $40 thousand, between $40–55 thousand, $55–75 thousand, and $75 thousand or more per year. The histological subtypes of melanoma considered in this study are superficial spreading, lentigo maligna, nodular, and acral lentiginous melanoma. Region, a variable consisting of five categories, denotes the patients’ county and bordering county’s general population size. These categories consist of counties in metropolitan areas of greater than or equal to 1 million, counties in metropolitan areas of 250,000 to 1 million, counties in metropolitan areas of less than 250 thousand, nonmetropolitan counties adjacent to a metropolitan area, and nonmetropolitan counties not adjacent to a metropolitan area. Patients’ ages were originally categorized into 19 groups, which were reduced to the following five groups: 0–29, 30–44, 45–59, 60–74 and 75 years of age or above. Sex is a binary variable consisting of males and females.

A total of 27,532 U.S. melanoma patients of whom 15,527 were males and 12,005 were females. The Hypertabastic Accelerated Failure Time model [21–26] was used to analyze the survival probabilities in patients with cutaneous or mucosal melanoma.

## Results

Table 1 gives the frequency, percentage, and median survival time (MST) of melanoma patients. Over 56% of individuals were male with a MST of 140 months. Among melanoma types, over 99% had cutaneous melanoma with MST of 149 months. Among histological subtypes, 66.9% had superficial spreading melanoma, followed by lentigo maligna melanoma (17.2%), nodular (13.9%), and acral lentiginous (2.0%). The MST for superficial spreading melanoma was 160.0 months, which was the highest MST among all histological subtypes followed by lentigo maligna melanoma (MST = 133.0 months). The least MST among histological subtypes belonged to nodular melanoma (85.5 months). With respect to age, individuals 45–59 years of age had the highest representation (29.2%); and the lowest MST belonged to those 75 years and older with a MST of 66 months. With regard to stage of the disease, 91.2% had localized (MST = 152 months), 7.8% had regional (MST = 71 months), and 1.0% had distant melanoma (MST = 11 months). Approximately 98.6% of our cohort were comprised of White (MST = 148 months). Individuals living in counties in metropolitan areas of greater than or equal to 1 million population (MST = 153 months) had the highest MST, representing only 45.5% of our cohort.

**Table 1:** Frequency, percent frequency and median survival time of melanoma patients.

Sex	Frequency	Percentage	MST (Month)
Female	12005	43.6	159
Male	15527	56.4	140
**Age (Years)**
0–29	1344	4.9	178
30–44	4655	16.9	182
45–59	8049	29.2	170
60–74	7720	28.0	143
75+	5764	20.9	66
**Histological Subtype**
Superficial Spreading Melanoma	18422	66.9	160
Lentigo Maligna Melanoma	4727	17.2	133
Nodular Melanoma	3832	13.9	85.5
Acral Lentiginous Melanoma	551	2.0	117
**Stage**
Distant	276	1.0	11
Regional	2154	7.8	71
Localized	25102	91.2	152
**Race**
Non-White	383	1.4	134
White	27149	98.6	148
**Income**
Less than $40,000	89	0.3	118
$40,000 –less than $55,000	2300	8.4	131
$55,000 –less than $75,000	13124	47.7	146
more than $75,000	12019	43.7	158
**Region**
Counties in metropolitan areas of greater than or equal to 1 million population	12526	45.5	153
Counties in metropolitan areas of 250,000 to 1 million population	7241	26.3	146
Counties in metropolitan areas of less than 250 thousand population	3300	12.0	151
Nonmetropolitan counties adjacent to a metropolitan area	2301	8.4	138
Nonmetropolitan counties not adjacent to a metropolitan area	2164	7.9	139
**Melanoma Type**
Mucosal	117	0.4	34
Cutaneous	27415	99.6	149

### Analysis of histological subtypes

As shown in Table 2, among patients who died, 44.7% had superficial spreading melanoma, followed by nodular melanoma (40.6), lentigo maligna melanoma (9.7%), and acral letiginous melanoma (4.9%). Additionally, 62.8% of those who died had localized, 30.6 had regional, and 6.6% had distant melanoma. Of those that had distant melanoma, 76.8% died; whereas 46.0% of those with regional, and only 8.1% of those with localized melanoma died.

**Table 2:** Estimated conditional probability of categories of melanoma variables.

	M	F	0–29	30–44	45–59	60–74	75+	<40k	40-<55k	55-<75k	75k+	SSM	LMM	NM	ALM
M			34.3	43.5	54.8	64.7	63.2	60.7	56.6	55.7	57.0	52.6	68.4	61.1	46.1
F			65.7	56.5	45.2	35.3	36.8	39.3	43.4	44.3	43.0	47.4	31.6	38.9	53.9
0–29	3.0	7.4						5.6	5.3	5.8	3.8	6.4	0.2	3.9	2.7
30–44	13.0	21.9						7.9	13.3	17.3	17.3	21.6	3.6	11.1	13.1
45–59	28.4	30.3						28.1	26.2	29.0	30.1	32.9	18.8	25.8	21.8
60–74	32.2	22.7						34.8	30.2	27.7	28.0	25.1	39.0	28.1	32.8
75+	23.5	17.7						23.6	25.0	20.3	20.8	14.1	38.4	31.1	29.6
<40k	0.3	0.1	0.4	0.2	0.3	0.4	0.4					0.3	0.2	0.5	1.1
40-<55k	8.4	8.3	9.0	6.6	7.5	9.0	10.0					8.5	7.1	9.7	6.2
55-<75k	47.1	48.4	56.3	48.7	47.3	47.0	46.3					48.0	48.7	45.3	44.3
75k+	44.2	43.0	34.4	44.6	44.9	43.6	43.4					43.3	44.0	44.5	48.5
SSM	62.5	72.7	87.1	85.8	75.2	59.9	45.0	62.9	67.7	67.4	66.3				
LMM	20.8	12.5	0.6	3.7	11.0	23.9	31.5	10.1	14.6	17.5	17.3				
NM	15.1	12.4	11.2	9.2	12.3	13.9	20.7	20.2	16.2	13.2	14.2				
ALM	1.6	2.5	1.1	1.5	1.5	2.3	2.8	6.7	1.5	1.9	2.2				
D	1.2	0.7	0.4	0.7	0.9	1.2	1.2	2.2	1.2	1.0	1.0	0.4	0.3	4.5	4.5
R	8.6	6.9	8.2	6.9	7.2	7.4	9.9	10.1	8.6	7.5	8.0	4.6	1.8	28.5	24.0
L	90.2	92.4	91.4	92.4	91.9	91.4	88.9	87.6	90.3	91.5	91.0	95.1	97.9	67.0	71.5
CMG1M	45.5	45.5	37.5	49.5	48.0	43.6	43.2	0.0	1.2	27.2	74.3	43.8	52.9	43.6	50.8
CM250k-M	26.7	25.7	30.4	25.9	25.0	27.3	26.2	1.1	5.0	34.6	21.5	27.1	24.4	24.6	26.5
CML250K	11.6	12.5	16.7	11.9	12.2	11.4	11.4	0.0	14.0	22.3	0.4	13.1	7.8	12.1	9.3
NMCAM	8.4	8.4	8.2	7.0	7.2	9.1	10.1	75.3	34.7	8.3	2.9	8.1	7.4	11.1	6.2
NMCNAM	7.9	7.9	7.3	5.8	7.6	8.7	9.0	23.6	45.1	7.6	0.9	7.8	7.5	8.6	7.3
W	98.8	98.4	98.6	98.6	98.8	98.5	98.5	95.5	99.3	99.1	98.0	99.1	99.3	98.0	82.0
NW	1.2	1.6	1.4	1.4	1.2	1.5	1.5	4.5	0.7	0.9	2.0	0.9	0.7	2.0	18.0
Mucosal	0.1	0.9	0.6	0.1	0.3	0.4	0.8	0.0	0.6	0.4	0.4	0.3	0.0	1.8	0.2
Cutaneous	99.9	99.1	99.4	99.9	99.7	99.6	99.2	100.0	99.4	99.6	99.6	99.7	100.0	98.2	99.8
Dead	14.0	8.8	6.0	7.4	9.9	13.3	17.1	22.5	14.2	11.2	11.8	7.9	6.6	34.3	29.0
Alive	86.0	91.2	94.0	92.6	90.1	86.7	82.9	77.5	85.8	88.8	88.2	92.1	93.4	65.7	71.0

	D	R	L	CMG1M	CM250k-M	CML250k	NMCAM	NMCNAM	W	NW	Mucosal	Cutaneous	Dead	Alive
M	70.3	61.7	55.8	56.4	57.3	54.5	56.4	56.4	56.5	49.1	9.4	56.6	67.2	55.0
F	29.7	38.3	44.2	43.6	42.7	45.5	43.6	43.6	43.5	50.9	90.6	43.4	32.8	45.0
0–29	2.2	5.1	4.9	4.0	5.6	6.8	4.8	4.5	4.9	5.0	6.8	4.9	2.5	5.2
30–44	12.0	14.9	17.1	18.4	16.6	16.8	14.1	12.4	16.9	16.7	4.3	17.0	10.7	17.7
45–59	27.5	26.9	29.5	30.8	27.8	29.7	25.3	28.1	29.3	25.1	22.2	29.3	24.7	29.8
60–74	32.6	26.6	28.1	26.9	29.1	26.7	30.4	31.0	28.0	30.8	25.6	28.1	31.7	27.6
75+	25.7	26.5	20.4	19.9	20.9	20.0	25.4	23.9	20.9	22.5	41.0	20.8	30.4	19.7
<40k	0.7	0.4	0.3	0.0	0.0	0.0	2.9	1.0	0.3	1.0	0.0	0.3	0.6	0.3
40-<55k	9.8	9.1	8.3	0.2	1.6	9.8	34.7	47.9	8.4	4.4	12.0	8.3	10.1	8.1
55-<75k	46.7	45.8	47.8	28.5	62.7	88.9	47.3	45.8	47.9	30.8	41.9	47.7	45.6	47.9
75k+	42.8	44.7	43.6	71.3	35.7	1.4	15.1	5.3	43.4	63.7	46.2	43.6	43.7	43.6
SSM	23.9	39.1	69.8	64.5	69.0	73.3	64.8	66.5	67.2	45.4	41.0	67.0	44.7	69.9
LMM	5.1	4.0	18.4	20.0	15.9	11.2	15.2	16.5	17.3	9.1	0.0	17.2	9.7	18.2
NM	62.0	50.7	10.2	13.3	13.0	14.0	18.5	15.2	13.8	19.6	58.1	13.7	40.6	10.4
ALM	9.1	6.1	1.6	2.2	2.0	1.5	1.5	1.8	1.7	25.8	0.9	2.0	4.9	1.6
D				0.9	1.1	1.0	0.9	1.2	1.0	4.4	14.5	0.9	6.6	0.3
R				7.7	8.1	6.8	8.6	8.3	7.7	20.1	30.8	7.7	30.6	4.8
L				91.4	90.7	92.3	90.6	90.6	91.4	75.5	54.7	91.3	62.8	94.9
CMG1M	42.8	44.8	45.6						45.5	43.9	45.3	45.5	43.1	45.8
CM250k-M	29.3	27.4	26.2						26.2	35.5	25.6	26.3	27.1	26.2
CML250K	11.6	10.4	12.1						12.1	6.0	8.5	12.0	11.5	12.1
NMCAM	7.2	9.1	8.3						8.4	3.4	10.3	8.3	10.3	8.1
NMCNAM	9.1	8.3	7.3						7.8	11.2	10.3	7.8	8.0	7.8
W	93.8	96.4	98.8	98.7	98.1	99.3	99.4	98.0			91.5	98.6	97.6	98.7
NW	6.2	3.6	1.2	1.3	1.9	0.7	0.6	2.0			0.0	1.4	2.4	1.3
Mucosal	6.2	1.7	0.3	0.4	0.4	0.3	0.5	0.6	0.4	2.6			2.2	0.2
Cutaneous	93.8	98.3	99.7	99.6	99.6	99.7	99.5	99.4	99.6	97.4			97.8	99.8
Dead	76.8	46.0	8.1	11.1	12.1	11.2	14.4	12.0	11.6	20.4	59.8	11.5		
Alive	23.2	54.0	91.9	88.9	87.9	88.8	85.6	88.0	88.4	79.6	40.2	88.5		

M = Male; F = Female; 0–29, 30–44, 45–59, 60–74, 75+ refer to Age Group; <40K, 40-<55K, 55-<75K, 75K+ refer to Income Group; SSM = Superficial Spreading Melanoma; LMM = Lentigo Maligna Melanoma; NM = Nodular Melanoma; ALM = Acral Lentiginous Melanoma; D = Distant; R = Regional; L = Local; CMG1M = Metro 1 Mil Pop; CM250K-M = Metro 250K-1Mil Pop; CML250K = Metro less than 250K Pop; NMCAM = Non Metro Adjacent to Metro; NMCNAM = Non Metro County Not Adjacent to Metro; W = White; NW = Non-White; Mucosal = Mucosal Melanoma; Cutaneous = Cutanious Melanoma; Dead = Dead of Melanoma; Alive = Alive or Dead due to Other Causes. Percentages listed in the table are represented conditioned on column label grouping, and are shaded according to percentage.

Among those who were diagnosed with nodular melanoma, 34.3% died. Given a patient was diagnosed with acral letiginous melanoma, 29.0% died. For patients with superficial spreading melanoma, 7.9% died. Approximately 6.6% of patients diagnosed with lentigo malignant melanoma died.

Of those who had mucosal melanoma, 59.8% died; but among cutaneous melanoma, the death rate was 11.5%. Among those who had distant melanoma, 62.0% had nodular melanoma. And only 5.1% had lentigo maligna melanoma. For patients diagnosed with localized melanoma, only 1.6% had acral lentigo melanoma and 69.8% had superficial spreading melanoma.

Approximately 0.4% of Whites had mucosal melanoma, but for non-whites, the percentage was 2.6%. Given a patient was diagnosed with mucosal melanoma, 58.1% had nodular melanoma and 0.0% had lentigo malignant melanoma. Of those who had cutaneous melanoma, 2.0% had acral lentigo melanoma and 67% had superficial spreading melanoma.

An examination of the distribution of histological subtypes indicates that superficial spreading melanoma had the highest frequency (66.9%), followed by lentigo maligna melanoma (17.2%) the lowest percentage belonged to acral lentiginous melanoma (2.0%). Histological subtypes played an important role in the survival probability of melanoma patients (*P* < 0.001). Among patients with superficial spreading melanoma, the death rate was 7.9% with a MST of 160 months. For those diagnosed with lentigo maligna melanoma, the death rate was 6.6% and the MST was 133 months. The MST for nodular melanoma patients was 85.5 months. Patients diagnosed with nodular melanoma had the highest percentage of death among all histological subtypes (34.3%). The percentage of death among patients with acral lentiginous melanoma was 29.0% which was the second highest percentage of death among all histological subtypes.

**Fig 1:**
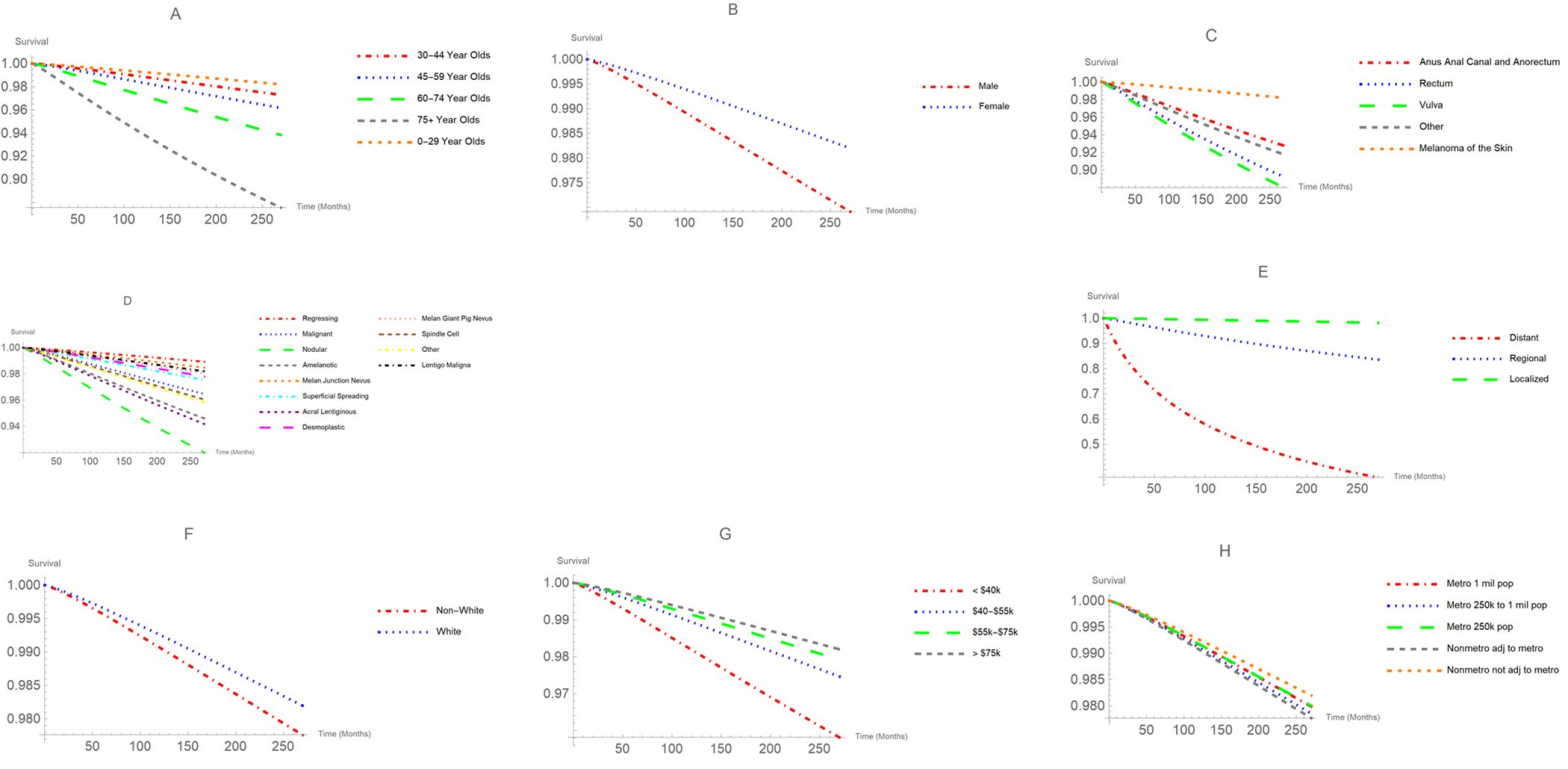
Survival probability curves for model variables.

**Fig 2:**
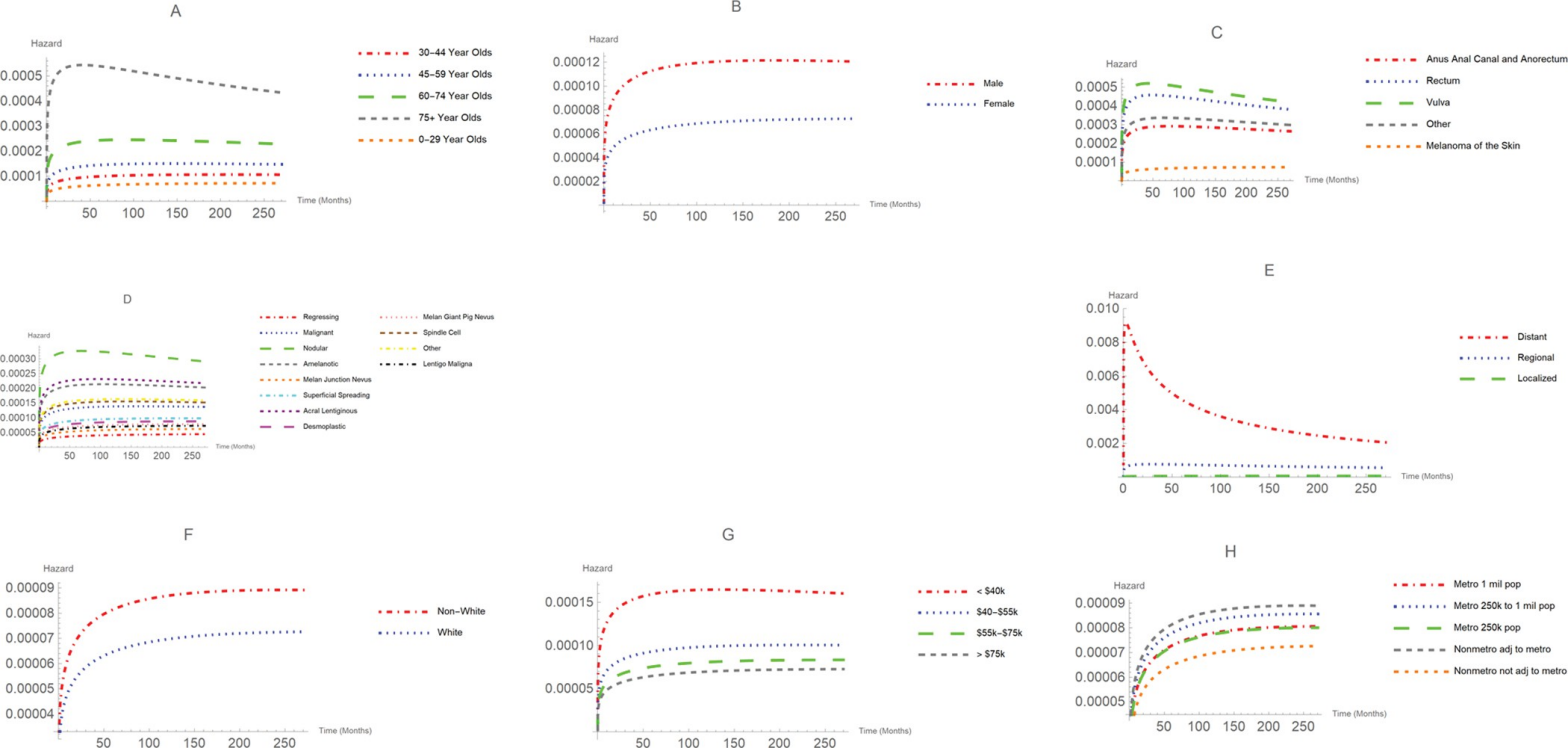
Hazard curves for model variables.

Figs 1 and 2 represent the survival probabilities and hazard (failure rate) curves, respectively. As indicated in Fig 1C, patients with lentigo maligna had the highest survival probability and nodular melanoma patients had the lowest survival probability. An interesting hazard shape can be seen in Fig 2D. At early stages of the disease, the hazard for distant melanoma sharply rises to its peak and gradually descends. While this phenomenon is not observed for localized and regional melanomas.

**Fig 3:**
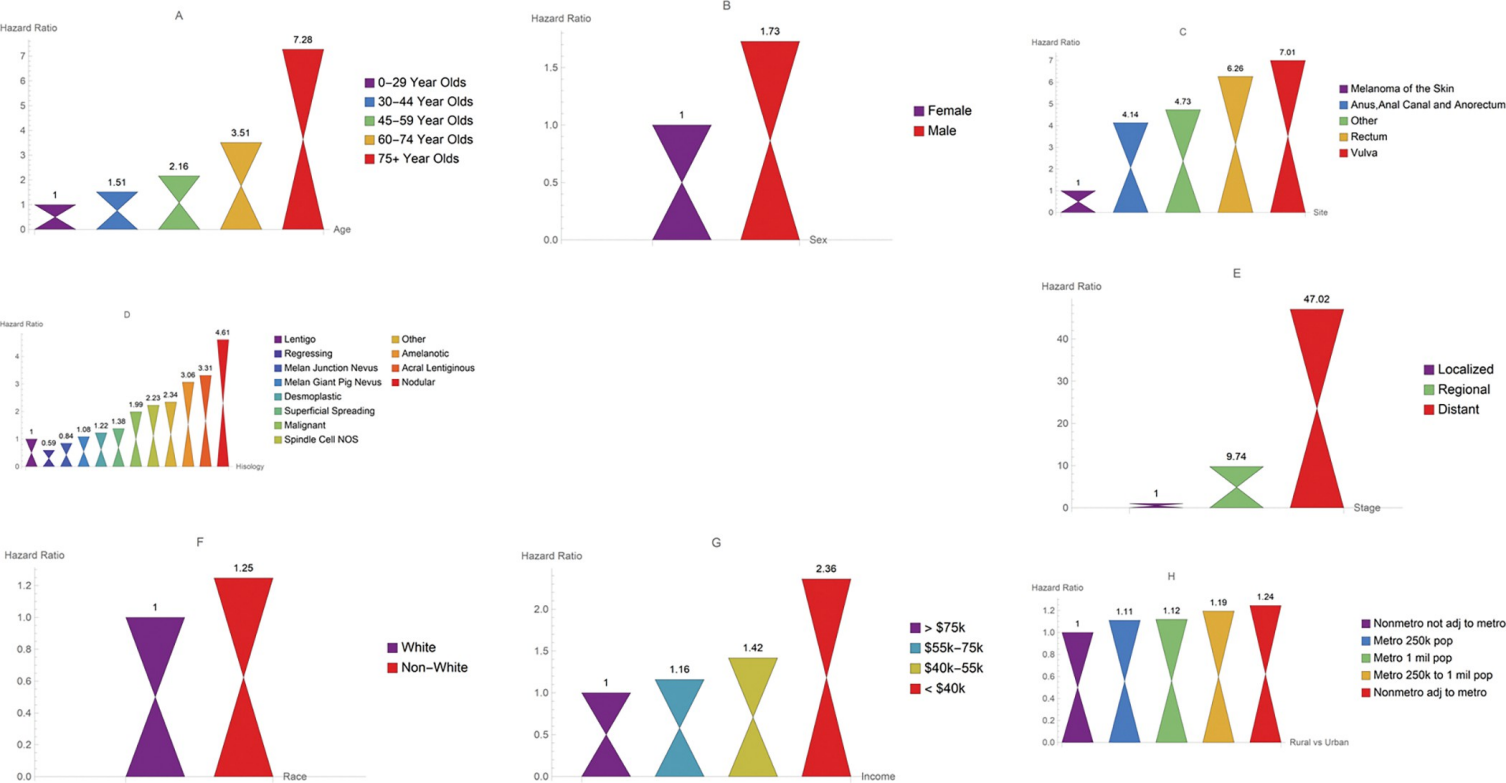
Bar graphs for the hazard ratios.

Using lentigo maligna melanoma as reference category, the HR listed in Fig 3C for nodular was the highest (HR: 3.40, 95% CI: 2.94–3.94) followed by acral lentiginous (HR: 2.87, 95% CI: 2.24–3.67), superficial spreading (HR: 1.33, 95% CI:1.17–1.51). Table 3 illustrates the MST between males and females. Across all histological subtypes, males had shorter MSTs than females.

**Table 3:** Median survival time of histological subtypes based on sex.

Histological subtype	Male	Female
**Nodular Melanoma**	72	121
**Lentigo Maligna Melanoma**	130	138
**Superficial Spreading**	153	168
**Acral Lentiginous Melanoma**	89.5	133

### Analysis of survival by age group

Age was a significant factor in the survival of melanoma patients (*P* < 0.001). The most frequent age group was 45–59, followed closely by those 60–74 years of age. For very young patients (younger than 29 years), the MST was 178 months. For patients (ages, 30–44 years), MST was 182 months and for people in the age group 45–59, the MST was 170 months. Patients 60–74 had a MST of 143 months. For patients aged 75 and above had a MST of 66 months. People 75 and older had the lowest survival probability and highest hazard curve across all age groups as shown in Figs 1–3A.

Additionally, using youngest patients (0–29 years of age) as a reference in Fig 3A, the HR for patients 75 years of age or older is 3.68 (95% CI: 2.87–4.71), for 60-74-year-olds is 2.24 (95% CI: 1.76–2.86), for 45-59-year-olds is 1.61 (95% CI: 1.27–2.06), and for 30-44-year-olds is 1.29 (95% CI: 1.00–1.67).

### Analysis of survival by sex

The MST for females was 159 months, and for males, the MST was 140 months. There was a significant difference in survival times between males and females (*P* <0.001). As shown in Figs 1–3B, males had a lower survival probability and higher hazard curve than females (HR: 1.53, 95% CI: 1.41–1.67).

### Analysis of survival by race

African American/Blacks accounted for only 0.3%, Asian/Pacific Islanders consisted of 0.9%, and American Indian/Alaskan accounted for 0.2% of our patients’ data. These three racial groups were bundled as non-Whites. White patients had a MST of 148 months. There was no significant difference in survival time between Whites and non-Whites (*P* = 0.304). The MST for non-Whites was 134 months. As Figs 1–3E shows, non-Whites had a lower survival probability and higher hazard curve when compared with Whites (HR: 0.87, 95% CI: 0.64–1.18).

### Analysis of survival by Region

Region was not a statistically significant factor. As shown in Figs 1–3G, patients residing in non-metropolitan areas not adjacent to metropolitan areas had the highest survival probability and lowest hazard rate of any region. The hazard ratio of patients living in a non-metro adjacent to metro region compared to those living in a non-metro not adjacent to metro region (HR: 1.15, 95%CI: 0.95–1.39).

### Analysis of survival by stage of the disease

Stage of the disease was a significant prognostic factor (*P* < 0.001). Patients with localized melanoma accounted for 91.2% of our cohort. The MST for localized patients was 152 months. The percentage of patients with regional melanoma was 7.8% with a MST of 71 months. Only 1.0% of patients had distant melanoma with a MST of only 11 months. Fig 1D illustrates the survival curves for different stage of melanoma. At any point in time, the lowest survival probability was associated with distant melanoma, while the highest survival probability belonged to individuals diagnosed with localized melanoma. The HR of distant melanoma to localized melanoma was 9.56 with a 95% CI: (7.58, 12.07). The HR for regional melanoma to localized melanoma was 4.01 with a 95% CI: (3.58, 4.50).

### Analysis of survival by income

Fig 1F shows the higher the income, the higher the survival probability. Only 0.3% of patients had income less than $40,000. The MST for people earning less than $40,000 was 118 months. For those 8.4% of patients who were making 40,000–55,000 dollars a year, the MST was 131 months. Patients in the income range of 55,000–75,000 constituted 47.7% of patients with the MST of 146 months. For those 43.7% of the patients making more than $75,000, the MST was 158 months. Those who were making below $40,000 were 59% more likely to die of the disease when compared with those individuals making more than $75,000. The HR for those who made below $40,000 to those made above $75,000 was 1.59 with a 95% CI: (0.88, 2.90).

As shown in Table 4, males diagnosed with distant nodular melanoma only had a 23% chance of surviving 5 years and a 12% chance of surviving 10 years.

**Table 4:** Five- and ten-year survival probabilities and respective 95% confidence intervals for white males and females age 45–59; living in metropolitan area with population of 1,000,000 or more: Diagnosed with melanoma of the skin; and income: 55-75k.

Sex	Histology	Localized		Regional		Distant	
		5-year	10-year	5-year	10-year	5-year	10-year
**Male**	**LMM**	0.98 (0.98, 0.98)	0.97 (0.96, 0.98)	0.88 (0.80, 0.96)	0.80 (0.70, 0.90)	0.60 (0.33, 0.87)	0.44 (0.17, 0.71)
	**NM**	0.91(0.90, 0.92)	0.83 (0.81, 0.85)	0.62 (0.58, 0.66)	0.46 (0.42, 0.50)	0.23 (0.15, 0.31)	0.12 (0.06, 0.18)
	**SSM**	0.98 (0.98, 0.98)	0.95 (0.95, 0.95)	0.84 (0.81, 0.87)	0.74 (0.70, 0.78)	0.52 (0.38, 0.66)	0.36 (0.23, 0.49)
	**ALM**	0.93 (0.89, 0.97)	0.87 (0.82, 0.92)	0.67 (0.56, 0.78)	0.52 (0.40, 0.64)	0.28 (0.07, 0.49)	0.16 (0.00, 0.33)
**Female**	**LMM**	0.99 (0.98, 1.00)	0.98 (0.97, 0.99)	0.93 (0.84, 1.00)	0.87 (0.75, 0.99)	0.71 (0.00, 1.00)	0.56 (0.00, 1.00)
	**NM**	0.94 (0.93, 0.95)	0.89 (0.87, 0.91)	0.72 (0.68, 0.76)	0.58 (0.53, 0.63)	0.34 (0.22, 0.46)	0.20 (0.10, 0.30)
	**SSM**	0.99 (0.99, 0.99)	0.97 (0.97, 0.97)	0.90 (0.87, 0.93)	0.82 (0.78, 0.86)	0.64 (0.41, 0.87)	0.48 (0.24, 0.72)
	**ALM**	0.96 (0.96, 0.96)	0.92 (0.88, 0.96)	0.77 (0.67, 0.87)	0.64 (0.52, 0.76)	0.40 (0.06, 0.74)	0.25 (0.00, 0.55)

For females diagnosed with distant nodular melanoma, the 5- and 10-year survival probabilities were 34% and 20% respectively. Another example, shown in Table 5, provides the five- and ten-year survival probabilities for White individuals in the age group 45–59, making between $55,000–75,000, living in metropolitan area with population of 1,000,000 or more, and diagnosed with the histological subtype nodular melanoma for males and females respectively. Males who were diagnosed with mucosal melanoma had the lowest survival probability regardless of the stage of the disease. The five- and ten-year survival probability for males with distant mucosal melanoma were 0.02 and 0.01 respectively. Females with distant mucosal melanoma have a five- and ten-year survival probability of 0.05 and 0.02, respectively.

**Table 5:** Five- and ten-year survival probabilities and respective 95% confidence intervals for males and females aged: 45–59; race: White; metro: Living in metropolitan area with population of 1,000,000 or more income: 55-75k; histology: Nodular melanoma.

Sex	Type	Localized		Regional		Distant	
		5-year	10-year	5-year	10-year	5-year	10-year
**Male**	**Cutaneous**	0.91 (0.91, 0.91)	0.83 (0.82, 0.84)	0.62 (0.59, 0.65)	0.46 (0.43, 0.49)	0.23 (0.17, 0.29)	0.12 (0.07, 0.17)
	**Mucosal**	0.60 (0.21, 0.99)	0.44 (0.04, 0.84)	0.19 (0.00, 0.57)	0.09 (0.00, 0.37)	0.02 (0.00, 0.29)	0.01 (0.00, 0.21)
**Female**	**Cutaneous**	0.94 (0.94, 0.94)	0.89 (0.88, 0.90)	0.72 (0.69, 0.75)	0.58 (0.55, 0.61)	0.34 (0.23, 0.45)	0.20 (0.10, 0.30)
	**Mucosal**	0.71 (0.59, 0.83)	0.57 (0.44, 0.70)	0.29 (0.13, 0.45)	0.16 (0.03, 0.29)	0.05 (0.00, 0.16)	0.02 (0.00, 0.09)

## Discussion

Histopathological subtypes play an important role in assisting health care providers be better informed when choosing treatment options for their melanoma patients [27]. Our research offers detailed insights into the survival of melanoma patients with respect to histological subtypes and their risk relationship with stage of the disease and melanoma type. In recent years, the overall improvement of melanoma patients’ survival probability noticeably started to improve in 2013 and this improvement appears to be primarily driven by a wave of new treatments such as immunotherapy or targeted therapies. The introduction of new therapies for distant melanoma was associated with a significant reduction in population-level mortality. Future research should focus on developing even more effective treatments, identifying biomarkers to select patients most likely to benefit, and renewing emphasis on public health approaches to reduce the number of patients with advanced disease [28].

Recent developments in understanding the molecular aspect of melanoma have helped the advancement of new and promising treatments. Precision medicine has significantly improved the treatment of distant melanoma patients. Targeted treatments have great potential in improving the survival of melanoma patients [29]. Amazingly, the hazard of distant melanoma was more than 9-fold higher when compared to localized melanoma. This indicates a significant influence of distant melanoma on the survival probability of patients. To increase the overall survivability of melanoma patients, efforts should be made to reduce the death rate among patients with distant melanoma by introducing new treatments. We also found a significantly higher risk of death in senior patients. The hazard for patients 75 or older was over three times higher when compared to the hazard of individuals 29 years of age or younger. Whites had a 14% higher hazard rate when compared to non-Whites. Lattanzi et.al. investigated biological and clinical variations between superficial spreading melanoma and nodular melanoma and the influence of histological subtypes in managing cutaneous melanoma [4].

The MST of patients with cutaneous melanoma was 149 months; whereas patients with mucosal melanoma had a MST of 34 months.

When we stratified the data by sex, the MST for males was shorter than females for all histological subtypes. The largest difference in the MST between males and females with respect to histological subtype was 49 months, which belonged to the nodular melanoma subtype. The smallest difference in the MST between males and females with respect to histology was 8 months which was associated with lentigo maligna subtype. Among histological subtypes, nodular melanoma had the shortest MST for both females and males (72 and 121 months) respectively. There was a sharp difference in MST for regional stage between females and males (90 months for females versus 61 months for males). The smallest difference in MST between females and males was associated with distant stage (13 months for females versus 11 months for males). The MST for females and males were 159 and 140 months respectively. Young females under 29 years of age had MST of 180 months but the MST for males in this age group was only 177 months. The MST for male and female seniors were 63 and 76 months respectively. Males making less than $40,000 had a MST of 109 months while for females the number was 124 months. For $75,000 or higher income bracket, female’s MST was 171 months while male’s MST was only 147 months. We hope that this paper will assist clinicians and health care providers in making wise decisions in the future treatment of melanoma patients by examining the risk associated with different combinations of age, race/ethnicity, sex, histological subtype, income, geographical region, melanoma type, and stage of disease. The strength of our study is the comprehensive assessment of the role of histological subtype and their relations with stage of the disease and melanoma type. It may play an important role in assisting both patients and health care providers to make more informed decisions about treatment strategy.

## Conclusion

In conclusion, single prognostic factors such as stage of the disease, age, gender, income, histological subtype, and melanoma type, as well as their combined effects, were the most influential prognostic factors in shaping the survival probability and hazard rates of melanoma patients. They also play a very important role in guiding clinicians and healthcare providers in their decision-making process regarding personalized treatment of their melanoma patients. Regardless of histological subtype, females had a uniformly higher survival probability than males. As we move into an era of personalized medicine and AI (artificial intelligence) these studies can be employed as a model to support oncologists to improve diagnosis and prognosis among melanoma patients. Examination of these histological subtypes and their link to diagnosis and prognosis among melanoma patients may have predictive valve and provide information that could lead to the development of novel and innovative therapies for the treatment of melanoma and other skin related cancers. Populations at higher risk for the most severe and aggressive subtypes of melanoma identified in this study should be targeted for programs that focus on education and awareness of melanoma and the benefits of early screening programs for skin cancer. The sex disparity observed in this study showing that for all histological subtypes, the survival probability for males was shorter than females. This could suggest that estrogen may have an overall protective value in the development and progression of all melanoma subtypes. We are aware that health equity is directly related to socioeconomic status that greatly influences social determinants of health (SDOH) and to achieve health equity we must provide medically underserved communities with education, awareness, routine screenings, and access to current treatment options for melanoma. The bundling of African American/Blacks Asian/Pacific Islanders American Indian/Alaskan as non-White racial groups performed in this study reflects the poor participation of some minority population in clinical studies. A sustained effort to increase the participation in minoritized populations in cancer clinical studies is essential for achieving health equity. Underserved populations are also likely to clinically present advanced stage disease, which reinforces the need for their inclusion in clinical trial studies and early screening programs. With the use of the R program found in the supplement, health care providers can estimate the patients’ survival probability based on personalized information and communicate patients’ chances of survival.

## Supporting information

S1 File (R)
